# Modelling Intra-Sinus Fluid Movements and Drainage Through Computational Fluid Dynamics Before and After Maxillary Sinus Augmentation: A Simulation-Based Pilot Study

**DOI:** 10.3390/jcm14010060

**Published:** 2024-12-26

**Authors:** İpek Necla Güldiken Sarıkaya, Alperen Tekin, Fatih Suda, Zeynep Gülen Çukurova Yilmaz, Mutlu Özcan

**Affiliations:** 1 Department of Dentomaxillofacial Surgery, Faculty of Dentistry, Istınye University, İstanbul 34010, Turkey; ipek.guldiken@istinye.edu.tr; 2 Department of Dentomaxillofacial Radiology, Faculty of Dentistry, Istanbul Medeniyet University, İstanbul 34720, Turkey; 3 Department of Dentomaxillofacial Surgery, Faculty of Dentistry, Graduate School of Health Sciences, Istanbul Medipol University, İstanbul 34810, Turkey; sudafatih95@gmail.com; 4 Department of Dentomaxillofacial Surgery, Faculty of Dentistry, Istanbul Medipol University, İstanbul 34810, Turkey; zeynepcukurova@gmail.com; 5 Clinic for Masticatory Disorders and Dental Biomaterials, Center for Dental Medicine, University of Zurich, 8006 Zurich, Switzerland; mutlu.ozcan@zzm.uzh.ch

**Keywords:** computational fluid dynamics, implant surgery, dental implants, maxillary sinus floor elevation, airway problems

## Abstract

**Objectives**: Sinus lifting, a procedure to augment bone in the maxilla, may cause complications such as sinusitis due to impaired drainage. This study aimed to assess how sinus lifting impacts airflow in the sinus cavity, which is essential for patients undergoing dental implants. Using computational fluid dynamics (CFD), this research analyzed airflow changes after sinus floor elevation, offering insights into the aerodynamic consequences of the procedure. **Methods**: Digital modeling and CFD analysis were performed using patient cone-beam computed tomography data. Three different sinus elevation scenarios, each with varying implant heights, were simulated. Airflow simulations were conducted to assess how reshaping the sinus cavity affects aerodynamics and airflow dynamics. Nasal resistance, calculated through pressure drops and flow rates, and wall shear stress, indicating potential mucosal damage, were evaluated. **Results**: Although some airflow changes occurred post-surgery, the implants primarily affected the front and rear of the elevated area, with little impact being seen on air entry points. **Conclusions**: Maxillary sinus lifting for dental implant placement may impair sinus drainage, especially at higher elevations, increasing the risk of mucosal damage due to intensified airflows in the reduced sinus volume. A more uniform, simplified intra-sinus structure may enhance fluid dynamics and reduce complications.

## 1. Introduction

Sinus augmentation is a surgical technique used to elevate the maxillary sinus floor to facilitate dental implant placement [1,2,3,4]. Despite its widespread use, the sinus lifting procedure is associated with a number of complications, including perforation of the Schneiderian membrane, acute maxillary sinusitis and chronic rhinosinusitis. Membrane perforation represents the most common complication, occurring in 20% to 25% of cases, with some studies noting rates as high as 42% [3]. The incidence of acute maxillary sinusitis has been reported to range from 10% to 20%, while the prevalence of symptoms consistent with chronic rhinosinusitis is estimated to be between 4% and 8% [5,6]. One of the complications that may arise following surgical elevation of the sinuses is sinusitis. This condition is typically the result of trauma to the sinus membrane during the surgical procedure itself. This injury can lead to an edema in the sinus lining, potentially obstructing the passage (ostium) connecting the sinus to the nasal cavity. The inflammation around the ostium can change the bacterial composition within the maxillary sinus, creating anaerobic conditions. With a reduced drainage capacity and the buildup of mucus, pathogens are unable to be effectively cleared, ultimately leading to sinusitis, which is a significant drawback of the sinus lifting procedure [4,7]. It is an inflammation of the sinus cavity’s mucous surface which leads to nasal congestion, purulent discharge and headaches, significantly affecting the patient’s quality of life [8].

The maxillary sinus is anatomically situated between the nasal and oral cavities, rendering it vulnerable to pathogens from either cavity and consequently making it the most frequently affected paranasal sinus with sinusitis [4]. The location of the ostium connecting the maxillary sinus with the nasal cavity is 25–35 mm above the sinus floor. According to some researchers, excessive graft material used to re-level the maxillary sinus floor can significantly decrease the sinus cavity volume and obstruct the ostium opening. This volume change may disrupt sinus airflow and drainage, resulting in various problems, including sinusitis [9,10].

Computational fluid dynamics (CFD) is a subfield of fluid mechanics that employs computer-based numerical analysis and data structures to model and simulate fluid flow and its interactions with boundary surfaces specified by defined boundary conditions [11]. CFD is a numerical analysis tool applied in various medical fields. In maxillofacial surgery, CFD has been used to predict post-surgical airway alterations [12,13,14,15,16,17,18]. However, its application to assess intra-sinus air pressure, fluid dynamics and postoperative complications after sinus lifting remains underexplored.

The primary aim of the study was to utilize CFD modeling to gain insights into the impact of sinus lifting on airway dynamics, especially in the maxillary sinus. By doing so, it seeks to provide valuable information for surgical planning, reducing the risk of complications. Ultimately, the study aims to predict and mitigate potential issues associated with sinus lifting procedures [19,20].

## 2. Material and Methods

### 2.1. Digital Modeling

The study was approved by the ethical committee of the School of Dentistry, Istanbul Medipol University (10840098-604.01.01-19315). The digital modeling of the maxillary sinus was generated using data obtained from a male patient aged 25–35 years who was in good overall health. This patient had missing maxillary molars and insufficient bone height for placing a dental implant in the edentulous posterior area. No signs of sinus inflammation or pathology were detected during the radiographic and clinical examination. The patient provided informed consent for the acquisition, modification and analysis of his radiological data for a simulational surgical study.

**Figure 1:**
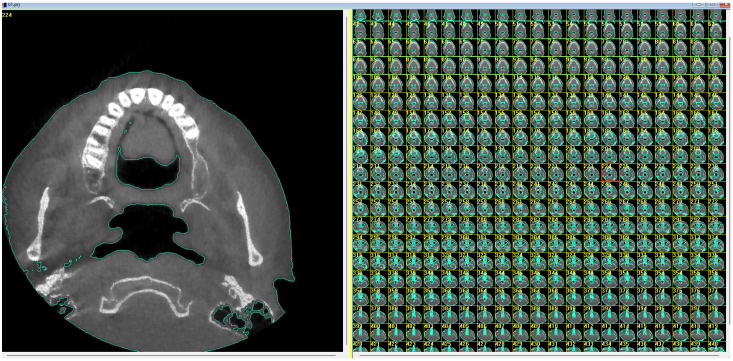
Three-dimensional soft tissue and airway modeling with CBCT and segmentation.

The cone-beam tomography data files were used for the modeling with the following parameters: 120 kV, 5 mA, exposure time 5 s, field of view of 17 × 23 cm, spatial resolution of 640 × 640 pixels, 448 cross-sectional slices, and voxel size 0.25 mm. The DICOM formatted data were imported into the i-CAT Vision software (Q version 1.8.1.10, Imaging Sciences International, Hatfield, PA, USA). Within i-CAT Vision software, the images representing the maxillary sinuses were extracted and segmented to create a three-dimensional (3D) airspace model (Figure 1).

Subsequently, the reconstructed stereolithography (STL) model was imported into Autoshaper (version 1.2), a software developed by AUTOMAPKI in Rue Saint Georges 97, 1050 Brussels, Belgium. In Autoshaper, surface extraction and conversation into a solid model were performed. A computer-aided design software, specifically Rhinoceros (version 5.0) from the US, was employed to simulate sinus augmentation. A computer simulation was designed to replicate a classical sinus floor elevation procedure at three distinct augmentation levels. The purpose of these three scenarios was to compare the aerodynamic changes in sinus cavities resulting from different extents of sinus membrane elevation and subsequent bone grafting. All scenarios began with an initial CBCT-measured residual bone height of 4 mm prior to augmentation. The implant placement and final bone heights in each scenario were as follows:

The three scenarios in the sinus lifting simulation were designed as follows:

Scenario 1:

Measured residual bone height on CBCT: 4 mm.

Height gained through sinus lifting: 4 mm, achieving a total bone height of 8 mm.

Implant placed: 7 mm in length.

Scenario 2:

Measured residual bone height on CBCT: 4 mm.

Height gained through sinus lifting: 8 mm, achieving a total bone height of 12 mm.

Implant placed: 11 mm in length.

Scenario 3:

Measured residual bone height on CBCT: 4 mm.

Height gained through sinus lifting: 12 mm, achieving a total bone height of 16 mm.

**Figure 2:**
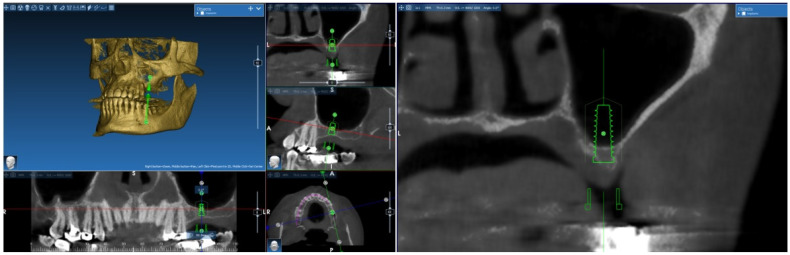
Simulational Surgery: Placement of Implants in the Maxillary Sinus of 4 mm—Bone Height.

Implant placed: 15 mm in length (Figure 2).

### 2.2. Mesh Designing

A tomographic image from the patient was converted into a three-dimensional model using a mesh-modeling technique by RealGuide software (Version 5.0, 3DIemme, Figino Serenza, Italy). Subsequently, simulations of sinus floor elevation surgeries across different vertical levels were performed on the same RealGuide platform.

The modeling in RealGuide software was performed using thresholds based on House-field Unit (HU) values obtained from the patient’s cone-beam computed tomography (CBCT) images in order to distinguish between the soft and hard tissues (gums, bone and teeth).

In the current study, the steps of the CFD analysis were performed as follows:

1. Generation of the geometric model for CFD numerical analysis from the CBCT image.
2. Formulation of the physical model in which the fluid flow phenomena can be defined.
3. Execution of the fluid flow simulation (using mathematical modeling and numerical computation methods).
4. Analysis of the simulation results.

The basic numerical equations of CFD have been describing as:

✓ Continuity equation (conservation of mass).
✓ Navier–Strokes or Reynolds-meaned Navier–Strokes equation.
✓ Energy equation (conservation of energy).
✓ Turbulence equation (in case of Reynolds-meaned Navier–Strokes equation).

The equations comprise closed, coupled, and nonlinear partial differential equations (PDEs) possessing over five unknown variables, including velocity components, pressure, temperature, and turbulence properties with a stochastic nature. Analytical methods cannot solve this PDE system except for the simplest cases. Thus, numerical methods are generally essential to addressing the problems.

The eddy viscosity definition undergoes modification with the SST turbulence model. This modification is imperative for accurately capturing the initiation of separation under pressure gradients. The calculation will involve solving the two equations formulated for turbulence modeling. One equation explains the transportation of turbulent kinetic energy (k) while the other defines the turbulent propagation velocity (ε) and turbulence (ω).

Autodesk CFD utilizes a combined solution algorithm with a basis-based, finite volume approximation and algebraic multigrid method. A residual error of second-order (<10^−3^) and instability in mass conservation (<10^−3^) were observed. Initial calculations were conducted on a nearly unstructured matrix for this purpose. The previously mentioned solution algorithm combined to guarantee fast and stable convergence rates.

**Figure 3:**
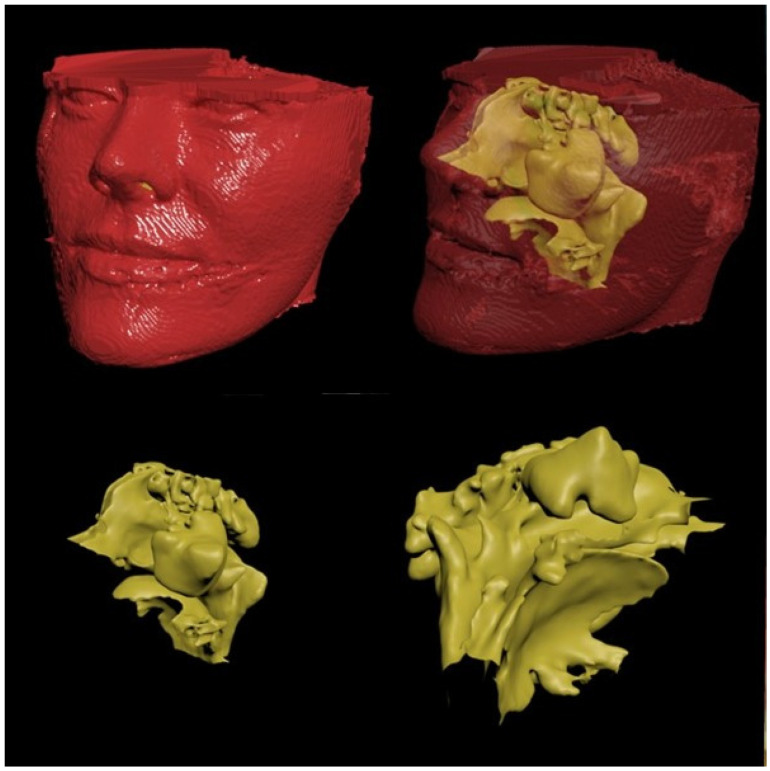
The preoperative 3D model of the maxillary sinus.

The objective of the geometric model was to obtain a non-intersecting 3D triangular model with a sufficient number of 3D and 2D meshes that cover all anatomical regions required for the study. The model under study was mathematical and consisted of a finite number of elements rather than a physical entity (Figure 3).

**Figure 4:**
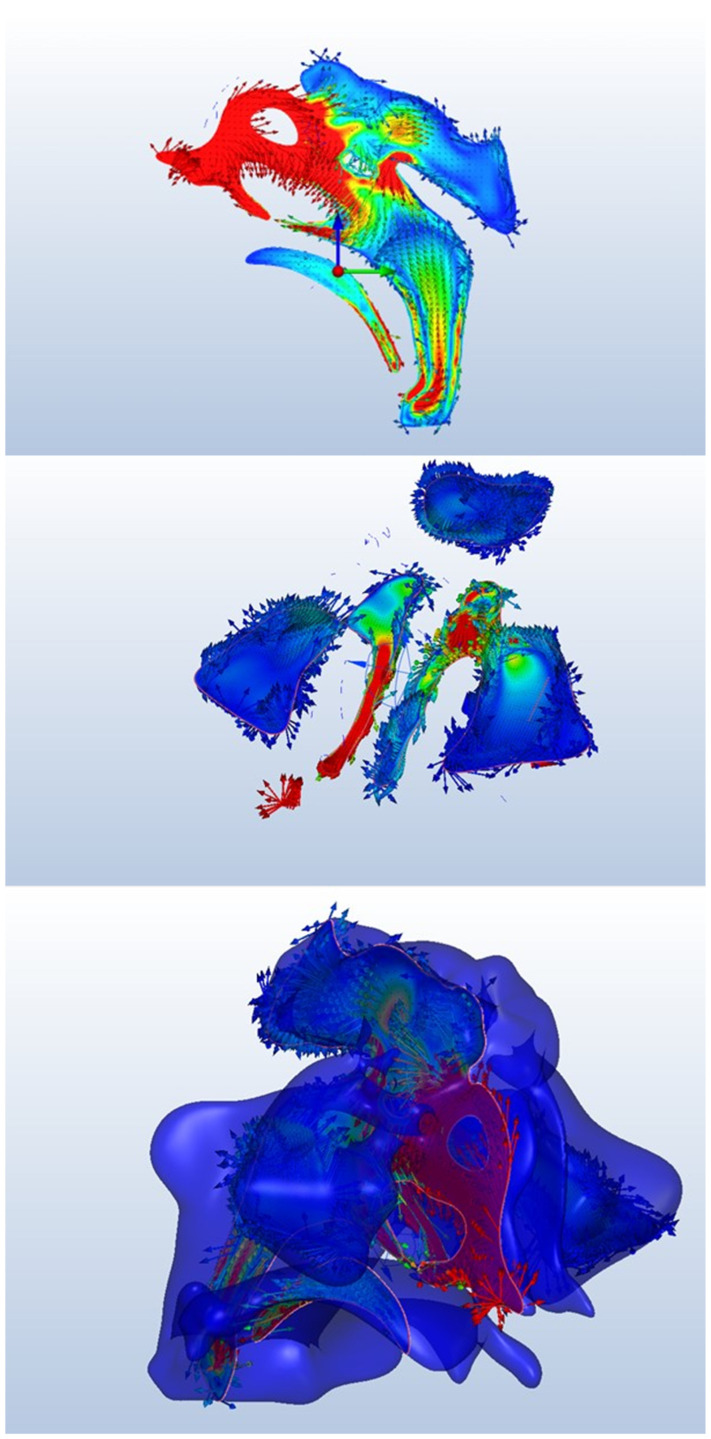
CFD analysis regarding the pressure differential in the maxillary sinus cavity prior to grafting.

Initially, a three-dimensional virtual model of the sinus cavity and its associated airways were created. During a normal resting respiratory cycle (inspiration–expiration), both laminar and turbulent flow were simulated. A smooth sinus wall, which spreads the mucous membrane lining the sinus cavity, was assumed to account for frictional flow. The air was modeled as an isothermal and incompressible medium with a density of 1.185 kg/m^3^ and a dynamic viscosity of 1.831 × 10^−5^ kg/(ms) at a temperature of 25 °C and an ambient pressure of 101.325 Pa (1 atm). The difference in air pressure between the inlet and outlet was determined to be 150 Pa. The calculation accounted for changes in pressure between the inlet and outlet of the airflow (nostrils and nasopharynx) and changes in the airflow, including pressure drop and flow rate, during inhalation and exhalation (Figure 4).

The models created using Rhinoceros were transferred to Autodesk CFD software (Version Ultimate 2021, Autodesk, San Francisco, CA, USA). Materials were assigned based on physical data within the scope of the study. Boundary conditions and fluid properties were defined, allowing for the necessary fluid mechanics analyses to be conducted.

## 3. Results

CFD analysis regarding the pressure differential in the maxillary sinus cavity prior to sinus floor elevation was conducted (Figure 4, Table 1 and Table 2).

**Figure 5:**
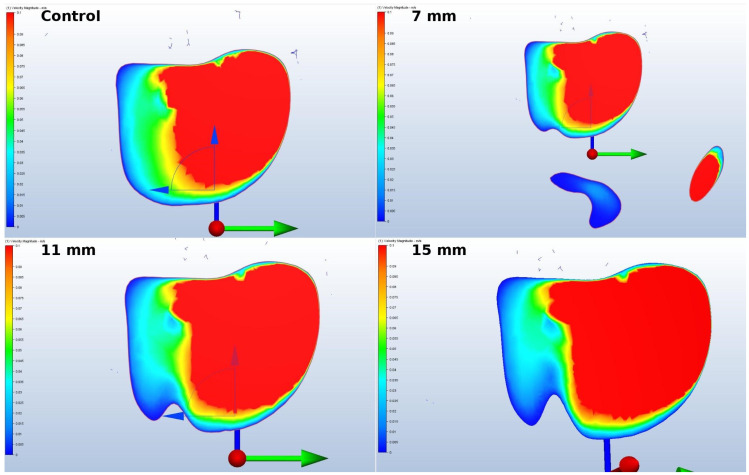
Nasal resistance.

Nasal resistance was defined as Pa·s/cm^3^, where is the pressure drop in pascals (Pa) and *Q* is the flow rate in milliliters per second (cm^3^/s), which was computed from the simulation results (Figure 5, Table 3).

**Figure 6:**
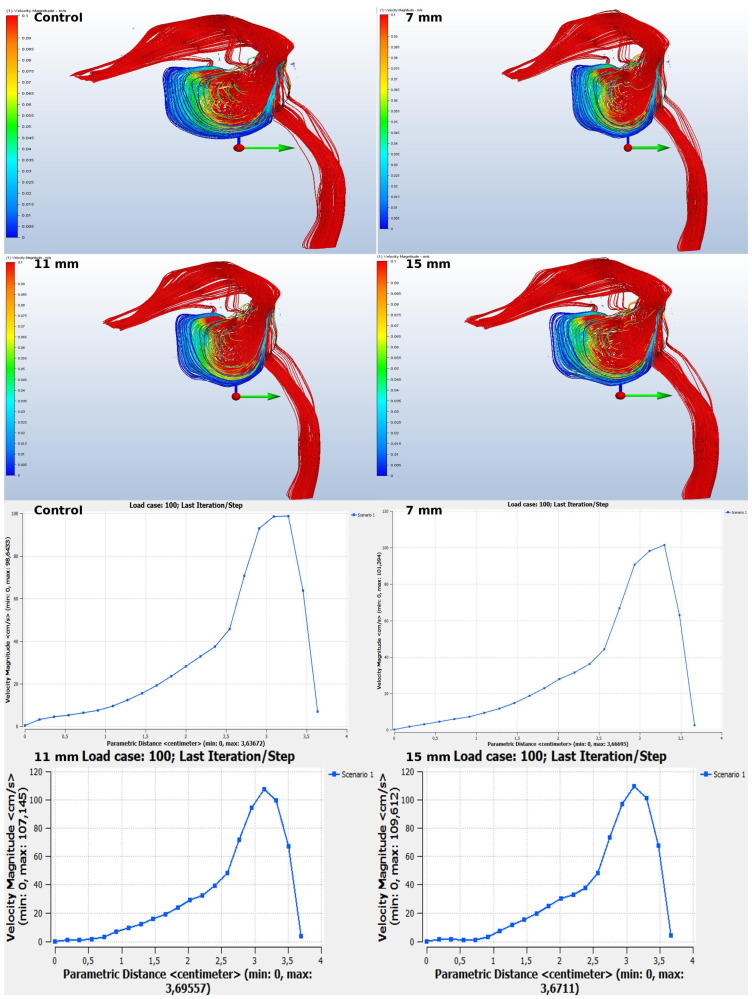
Airflow Streamlines in the Olfactory Area.

The diagram illustrates a marked reduction in flow rate in the location subsequent to the sinus lift. Despite this, the overall velocity increased, resulting in an elevated likelihood of harm to the sinus walls (Figure 6).

**Figure 7:**
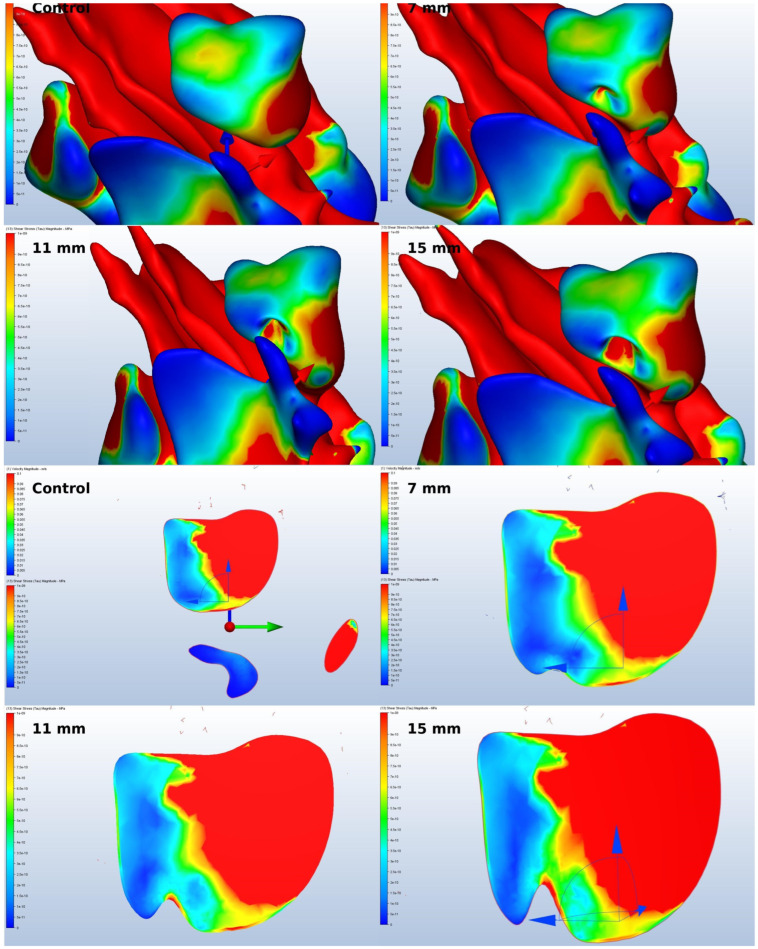
Values of mucosal wall shear stress.

Wall shear stress refers to the tangential drag force created by air moving over the surface of the mucosa, and it is determined by the velocity gradient of air near the mucosal surface. The increased wall-shear force had the potential to cause greater damage to the mucosa, as shown in Figure 7.

**Figure 8:**
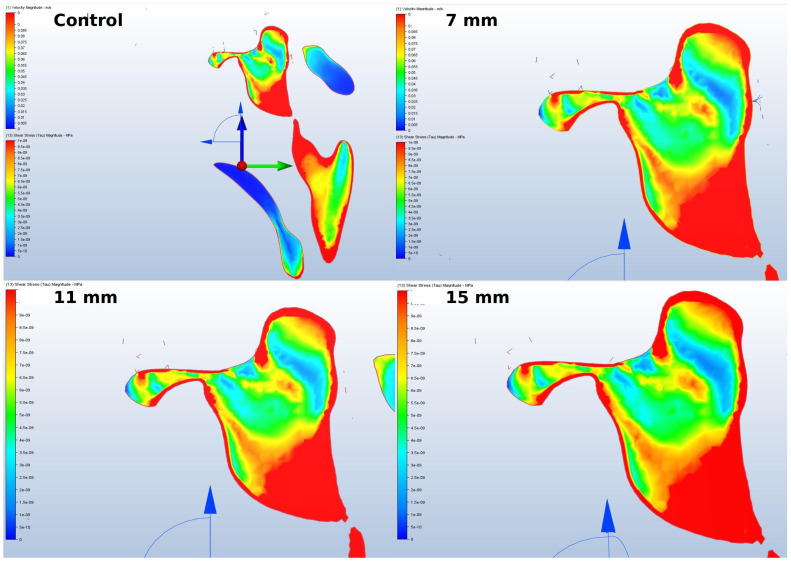
The evaluation of change in the air entry and exit channels of the sinus.

Although changes were observed in the sinus’s air entry and exit channels, no difference was formed. This most likely indicates that the implant anomaly probably affected the front and rear regions of the lifting more than the air entries. For airflow considerations, sinus lifting operations with larger surface areas seemed more successful (Figure 8).

## 4. Discussion

The anatomy of the maxillary sinus is believed to be linked to intra-sinus pathology [2]. Maxillary sinus elevation surgery is frequently performed to augment an inadequate alveolar bone volume beneath the sinus floor. This surgical procedure not only diminishes the volume of the maxillary sinus but also modifies its anatomy and physiology, resulting in various complications, including sinusitis, wound infection, abscess, graft exposure, or loss [21]. While physio-anatomical variations such as septal deviation, concha bullosa, the presence of the Haller cell, and nasal mucosa thickening due to nasal allergy may pose a high risk for postoperative sinusitis after sinus elevation, the relationship between the drainage system and anatomical variations other than the ostium and septa remains controversial [22,23,24]. In summary, the incidence of intra-sinus pathological conditions is related to the anatomy of the maxillary sinus. Specifically, our simulation results illustrated that this surgical modification effectively decreased flow in the cavity with a raised sinus floor, particularly in the posterior region of the elevated area.

The outcomes of our investigation aligned with prior research, underlining the significance of air and fluid movement in the sinus, the interconnection between the fluid dynamics and sinus anatomy and the impact on drainage in the sinus. In their retrospective study, Won Lee et al. explored the correlation between anatomical deviations that may hinder the sinus ostium and the likelihood of complications subsequent to the raising of the floor of the maxillary sinus. Patients with ostiomeatal variations, including nasal septum deviations, concha bullosa, a paradoxical curve in the middle turbinate, accessory ostia, and, particularly, Haller cells, experienced a higher incidence of postoperative sinusitis according to the studies [2,19]. The researchers attributed this to a delay in recovery following impaired sinus drainage. However, the number of articles examining this relationship in the literature is limited. The presented CFD analysis revealed that the formation of a stigma due to the elevated floor of the maxillary sinus, which protruded into the sinus cavity, hindered the flow in this area and operated similarly to the ostiomeatal variations observed in the study by Won Lee et al. [2]. This impeded flow may have the potential to compromise drainage and could contribute to certain postoperative complications. Another study investigated the correlation between the length of the infundibulum and the height of the ostium, which are variations in the anatomy of the maxillary sinus. The results showed that the presence of an intra-sinus septum increased as the ostium height increased, and this occurrence was more frequent in males [25]. However, the study suggested that conditions like septum deviation, concha bullosa, and Haller cells did not significantly impact the drainage system [24]. Since our research evaluated three distinct surgical simulation models in regard to a singular surgical model, we were unable to effectively evaluate any differences in regard to sinus drainage and fluid dynamics amongst various ostium anatomies.

Nonetheless, there was no significant difference in the flow rate or pressure in the ostium region postoperatively within the upper area of the sinus cavity. In brief, although variations in ostium anatomy may increase the likelihood of certain pathologies through the obstruction of sinus drainage, no fluid flow differences were observed in the ostiomeatal region following sinus lifting. The primary change after surgery occurred on the sinus floor, specifically in the surgical area where augmentation was performed. Thus, during the planning of sinus elevation surgery, emphasis should be placed on evaluating anatomical variations and irregularities close to the surgical site rather than focusing on the ostiomeatal complex anatomy. One reason might be that the airflow in and out of the ostium was much faster than the intra-sinus flow, which could hinder sinus mucosa clearance through drainage towards the bottom [26]. However, patients with sinus drainage complications due to variations in the ostium and its surroundings might face more risky situations in terms of possible complications. The authors of the presented study believed that this outcome may necessitate reassessing both the procedure of sinus elevation and the techniques of placing subsequent implants.

Another finding of the study that requires examination was the rise in the total flow rate in the sinus following the simulation surgeries we conducted, resulting in increased stress on the Schneiderian membrane lining the cavity. This factor can be interpreted as a risk factor for membrane damage. The increase in the risk is believed to be due to the sinus wall-shear stress in the flow direction, which increased in direct proportion to the amount of amplification. Choi and colleagues conducted a CFD study to evaluate the impact of sinus irrigations after maxillary sinus surgery. The study concluded that sinus flow accelerated and shear stress on the membrane increased following the surgery, and the findings were consistent with the presented study [10]. Similarly, Li et al.’s study [27], which closely mirrored our methodology, supported the notion that an increasing flow velocity heightened sinus wall shear stress.

As a deliberate distinction, while this study analyzed the effect of the shape change after sinus elevation on the airflow in the sinus, another study examined the effect of respiratory movements on the shape change at the grafted area after sinus elevation. According to Li et al., the mechanical impacts of respiratory functions on the new shape formed at the base after sinus elevation were negligible [27].

Creating a shape change as an elevation in the region where a single implant will be placed significantly affects airflow, even if it is a slight change in comparison to the general volume of the sinus, according to our study. Although both studies demonstrated that sinus floor elevation had a substantial impact on intra-sinus fluid dynamics, they approached the change from various perspectives. Thus, the results of these two studies appeared to be complementary and, together, offered a more comprehensive understanding of sinus floor elevation. In other words, simultaneously analyzing the amount of sinus elevation and respiratory movements seems necessary in order to comprehend the intra-sinus fluid dynamics.

This research was the inaugural study on the impact of the alteration in the sinus floor’s shape following a maxillary sinus lift on intra-sinus fluid dynamics. The authors have contended that this research is a significant contribution to the literature.

Clinical Recommendations:

In light of the findings of this study, a number of clinical considerations are proposed in order to minimize complications and optimize surgical outcomes during maxillary sinus elevation procedures.

Preoperative Assessment:

- A comprehensive assessment is essential for optimal surgical outcomes.
- Anatomical variations near the surgical site, such as intra-sinus septa or sinus floor deviations, should be evaluated. They can affect fluid dynamics and complications.
- Use CBCT imaging to identify and address ostium-related variations in patients with a history of sinus pathology or drainage issues.

Surgical Planning:

- Sinus floor elevation should be tailored to the patient’s anatomy to minimize disruption to sinus airflow.
- A gradual increase in augmentation height is important to avoid changes in sinus wall shear stress, which could affect the Schneiderian membrane.

Postoperative Management:

- Monitor patients for signs of poor sinus drainage after surgery, especially those with variations.
- Implement postoperative measures, such as saline irrigations, to support sinus clearance and reduce inflammation.

These recommendations aim to bridge the gap between computational findings and clinical practice, ensuring that surgical interventions are both evidence-based and patient-specific.

To address potential study limitations, CFD examines fluid mechanics in a three-dimensional space. Within the medical field, CFD analyzes physical factors such as pressure, speed and stress to investigate their physiological effects [11,12,13,14]. However, controversy surrounds the adequacy of this engineering approach to assessing the physiology of specific diseases or the potential impact of surgical procedures on the involved tissues. As with many pathophysiological conditions, the development of diseases following maxillary sinus surgeries is multifactorial. Through our study, we sought to examine the physical factors linked to surgery that could increase the risk of post-maxillary sinus elevation pathological conditions. However, it cannot be denied that various variables, including surgical procedure variations and patient-specific factors, apart from the morphology of the sinus cavity and its influence on intra-sinus fluid dynamics, play a role in the etiology of complications that may arise following the maxillary sinus elevation procedure [19,21,22,23,24,25]. In this regard, the presented study has only examined potential complications after maxillary sinus augmentation from a single perspective. One notable limitation of our study is that, despite employing various simulation models, the analysis was based on data from a single patient. To minimize the influence of any unique anatomical features on the results, we specifically selected a patient with no maxillary sinus pathology or anatomical variations, ensuring that the CBCT data provided well-defined and clearly discernible sinus boundaries. Although this approach enabled a controlled, pilot-level investigation, increasing the number of patients included in future research will be essential to enhancing the generalizability of the findings. Building on the insights gained here, more comprehensive CFD studies and empirical investigations involving larger patient cohorts will be needed to fully elucidate the implications of these results.

## 5. Conclusions

Sinus floor augmentation for dental implant placement affects fluid dynamics in the maxillary sinus. Proper intra-sinus fluid drainage is vital for sinus health and preventing postoperative complications. Identifying anatomical variations via CT scans before the surgery is essential. This simulation study analyzes postoperative airflow changes in the maxillary sinus through CFD simulations, emphasizing fluid dynamics in surgical planning. Increased sinus elevation may reduce airflow drainage, potentially causing sinus membrane stress and perforation risk. Intra-sinus septa and lifting procedures can yield similar effects. A uniform sinus anatomy benefits fluid dynamics and drainage, especially on a horizontal plane. CFD modeling provides an innovative approach to predicting potential postoperative sinus health complications and enables sinus lifting plans that are customized to an individual patient’s needs.

## Figures and Tables

**Table 1:** Element node numbers.

Element Node Numbers	Nodes	Elements
Control	183,384	594,660
7 mm	186,651	607,170
11 mm	188,045	612,577
15 mm	189,550	617,495

**Table 2:** Material information fluid air properties.

Material Information Fluid Air Properties	Value	Units
Reference Pressure	1.01325 × 10^6^	dyne/cm^2^
Reference Temperature	19.85 Celsius	Celsius
Gas Constant	2.8705 × 10^6^	cm^2^/s^2^·K
Viscosity Constant	0.0001817 p	poise
Conductivity Constant	0.0002563	W/cm·K
Compressibility Constant	1.4	-
Specific heat Constant	1.004	J/g·K
Emissivity Constant	1	-
Wall roughness Constant	0	centimeter

**Table 3:** Nasal resistance.

	7 mm	11 mm	15 mm
Total nasal resistance (Pa·s/cm^3^)	0.0000040119	0.0000025307	0.0000001106
Nasal resistance ratio (%)	100	63.07904874	2.755642913
Max Velocity in Sinus	101.394	107.145	109.612
Velocity Change Ratio	2.788532014	8.618628939	11.11955906

## Data Availability

The original contributions presented in the study are included in the article, further inquiries can be directed to the corresponding author.
